# Emerging Insights into the Roles of the Rhizome–Culm System in Bamboo Shoot Development through Analysis of Non-Structural Carbohydrate Changes

**DOI:** 10.3390/plants13010002

**Published:** 2023-12-19

**Authors:** Tianyi Hu, Linghui Kong, Sisi Hu, Meng Deng, Guangyao Yang, Qiang Wei, Fen Yu

**Affiliations:** 1 Jiangxi Provincial Key Laboratory for Bamboo Germplasm Resources and Utilization, Jiangxi Agricultural University, Nanchang 330045, China; hutianyi528@163.com (T.H.);; 2 State Key Laboratory of Tree Genetics and Breeding, Co-Innovation Center for Sustainable Forestry in Southern China, Bamboo Research Institute, Key Laboratory of National Forestry and Grassland Administration on Subtropical Forest Biodiversity Conservation, School of Life Sciences, Nanjing Forestry University, Nanjing 210037, China

**Keywords:** *Phyllostachys edulis*, bamboo shoot, culm, rhizome, parent bamboo, non-structural carbohydrates, starch granule

## Abstract

Non-structural carbohydrates (NSCs) required for bamboo shoot development, the critical stage that determines the yield of a bamboo stand, originate from the parent bamboo with the complex underground system. However, the metabolic mechanism of NSCs in the rhizome–culm system during bamboo shoot development remains unclear. In this study, we focused on the changes of NSCs in the rhizome–culm system and used anatomical, physiological, and biochemical methods to investigate the metabolism of NSCs in bamboo shoots of *Phyllostachys edulis* and the role of NSCs supply in the parent bamboo at different ages. The results showed that NSCs were accumulated and consumed from the bottom to the top in a bamboo shoot, which was consistent with the developmental pattern. The starch granules were stored in advance. The bamboo sheath stored starch from the dormant stage of shoot buds. The functions of culms and rhizomes showed age-dependent differences. Adult culms showed the highest capacity to provide NSCs, with more stored NSCs and higher β-amylase activity. Conversely, young culms seemed to prefer their growth, while old culms tended to store starch. Accordingly, adult rhizomes preferred sugar transport due to the lowest starch storage, lower ADP-glucose pyrophosphorylase (AGPase) activity, and higher β-amylase activity, while young and old rhizomes tended to prefer starch storage. These results provide a basis for further understanding of nutrient metabolism in bamboo stands.

## 1. Introduction

Bamboo is a sustainable “green material” with an important role in carbon sequestration and environmental remediation [1,2,3]. Bamboo shoots, with explosive growth, determine the yield of bamboo timber and the rise and fall of bamboo stands. Previous studies have shown that bamboo shoots can grow more than 1 m per day, producing 570 million cells per internode [4]. During this process, bamboo shoots require a large amount of material nutrients. Non-structural carbohydrates (NSCs), as direct products of photosynthesis, including soluble sugars and starch, are involved in plant metabolism and impact growth rhythms [5]. Some studies have pointed out that shoot sheaths have the potential for photosynthesis because of the complete photosynthesis-related genes and a large number of chloroplasts [6,7,8]. Chen et al. indicated that the shoot sheaths of *Bambusa multiplex* can even provide about 40% of the energy for the growth of bamboo shoots [9]. However, the limited photosynthesis of shoot sheaths was still insufficient to supply such rapid consumption of bamboo shoots before the leaves functioned, and more nutrients were proved to come from the parent bamboo, especially the culms, which were connected by rhizomes [10]. A steep increase in sap flow rate followed by a rapid decrease in NSC content was observed in the parent bamboo from shoot emergence until the end of branches and leaves growth in August, and the number and development of bamboo shoots were also correlated with the growth status of parent bamboo [10,11,12,13]. Li et al. found that the new shoots were more abundant in unfertilised plots by unevenly applying fertiliser to the moso bamboo stand [14]; this is due to the special “rhizome–culm” system of bamboo, a typical clone of the plant, which makes the whole bamboo stand connected like multiple branches of a tree [15]. Nutrients integrated physiologically in the rhizomes are transported to the ramets together with water, affected by branching and the environment [16,17,18,19,20]. The direction of transport of substances in rhizomes is bidirectional and related to source–sink relationships, either along the rhizomes or going through rhizomes, so that parent bamboos of every age may provide nutrients for bamboo shoots [17,21,22]. The source of nutrients in bamboo shoots during such rapid growth remains unknown until the influence of different age parent culms is clarified.

Little research has been conducted on the dynamics of NSCs in bamboo stand systems, mainly because of their complex rhizome–culm system [5,23,24,25]. Starch, the main storage form of non-structural carbohydrates, can be interconverted by enzymes with soluble sugar that can be used directly. In this process, ADP-glucose pyrophosphorylase (AGPase) is the key rate-limiting enzyme associated with starch synthesis in plants [26,27]; β-amylase is an exo amylase, and some studies have suggested that it plays a major role in starch degradation in bamboo [28,29]. Starch accumulates in the shoots in winter and is consumed in spring by converting it into soluble sugars [30]. Among all organs, the highest content of soluble sugars was found in bamboo leaves, and the highest content of starch was found in the culm stump [31,32]. The contents of starch and soluble sugars in bamboo culms aged over three years were higher in April and lower in May [31,33]. Related studies also reported that bamboo responded and recovered during environmental stress by adjusting the contents of starch and soluble sugars regulated by enzyme activities [34,35]. Furthermore, the metabolism of NSCs in bamboo depends on the transport of water and assimilates by the dense longitudinal vascular bundles in the internodes and the complex network of vascular bundles at the nodes, with the bamboo sheaths being the controllers [36,37,38]. The flow pattern of NSCs in the rhizome–culm system has not been reported, especially during the special period of bamboo shoot development. In addition, as the connecting pathway between different ramets, rhizomes have been studied mainly for their growth regulation, distribution pattern, and biomass allocation, with insufficient discussion on their function, and their ability to store NSCs is also controversial [39,40,41,42,43].

In the present study, we used the widely distributed and typical fast-growing *Phyllostachys edulis* as the material to investigate the dynamic changes of NSCs and the physiological mechanisms during the development of bamboo shoots. Our results demonstrate the functional differences between various age culms of parent bamboo and different age rhizomes. Additionally, we elucidate the regulatory mechanisms of starch and propose a possible supply pattern of NSCs in the rhizome–culm system during the development of bamboo shoots. This study can lead to a better understanding of material allocation in bamboo stands and shed light on the production and management of bamboo stands.

## 2. Results

### 2.1. Changes in Non-Structural Carbon Hydration of Shoots

**Figure 1:**
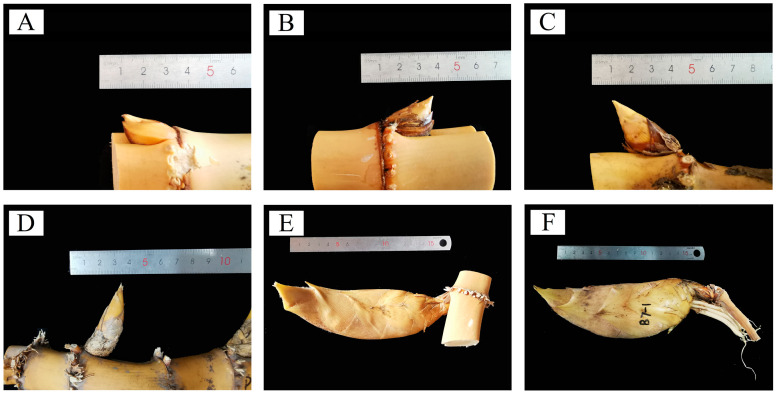
Morphological characteristics of bamboo shoot buds at different developmental stages in winter. (**A**) Dormant stage; (**B**) germination stage; (**C**) early developmental stage; (**D**) middle developmental stage; (**E**) late developmental stage; and (**F**) mature stage.

The development of underground bamboo shoot buds was divided into the dormant stage, the germination stage, the early developmental stage, the middle developmental stage, the late developmental stage, and the mature stage based on morphological characteristics and cellular structure (Figure 1).

**Figure 2:**
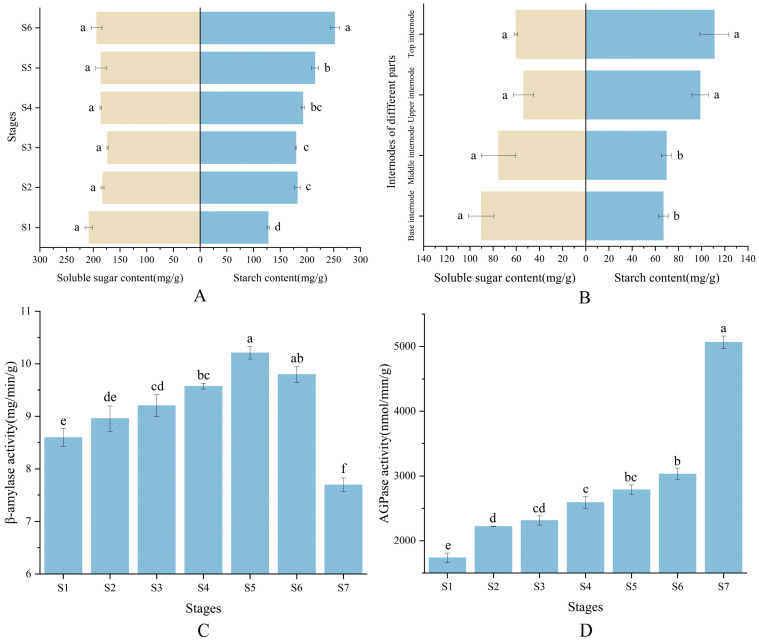
Changes in NSC content and starch-metabolising enzyme activities in bamboo shoots at different stages of bamboo shoots. (**A**) Content of NSCs in shoots of different developmental stages in winter; (**B**) content of NSCs in different internodes of bamboo shoots in spring; (**C**) β-amylase activity in the shoots of different developmental stages; (**D**) AGPase activity in the shoots of different developmental stages. S1, dormant stage; S2, germination stage; S3, early developmental stage; S4, middle developmental stage; S5, late developmental stage; S6, mature stage; and S7, elongation stage. Different letters in each graph indicate significant differences, *p* < 0.05.

The starch content in shoot buds exhibited a consistent increase, whereas soluble sugars initially decreased and subsequently experienced an increase. The AGPase activity in buds demonstrated a gradual increase, while the β-amylase activity initially rose and was followed by a slight decline (Figure 2). During the elongation growth of bamboo shoots, there was a significant decrease in the contents of starch and soluble sugars, while AGPase activity increased significantly (Figure 2).

**Figure 3:**
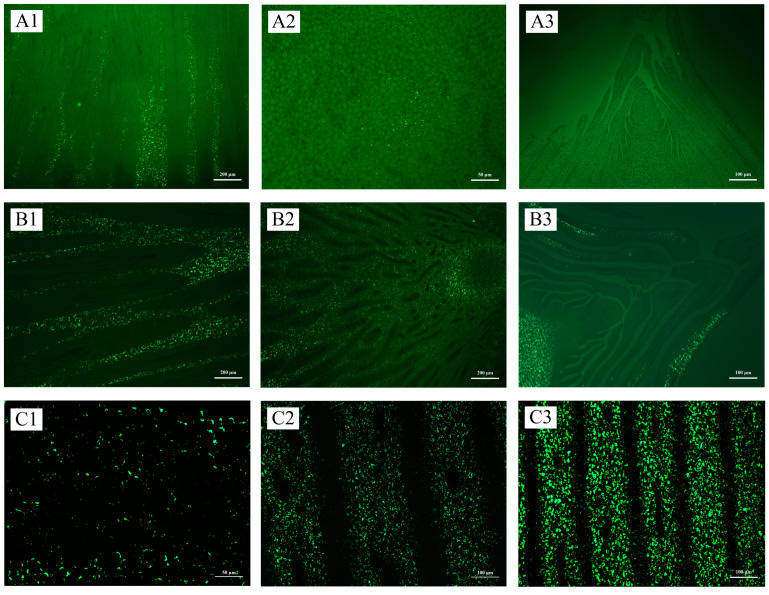
Starch granule distribution in winter shoot buds during the dormant stage, early developmental stage, and late developmental stage. (**A1**) The base of shoot buds at the dormant stage; (**A2**) the middle of bamboo shoots at the dormant stage; (**A3**) the upper of bamboo shoots at the dormant stage; (**B1**) the base of bamboo shoots at the early developmental stage; (**B2**) the middle of bamboo shoots at the early developmental stage; (**B3**) the upper of bamboo shoots at the early developmental stage; (**C1**) the base of bamboo shoots at the late developmental stage; (**C2**) the middle of bamboo shoots at the late developmental stage; and (**C3**) the upper of bamboo shoots at the late developmental stage.

**Figure 4:**
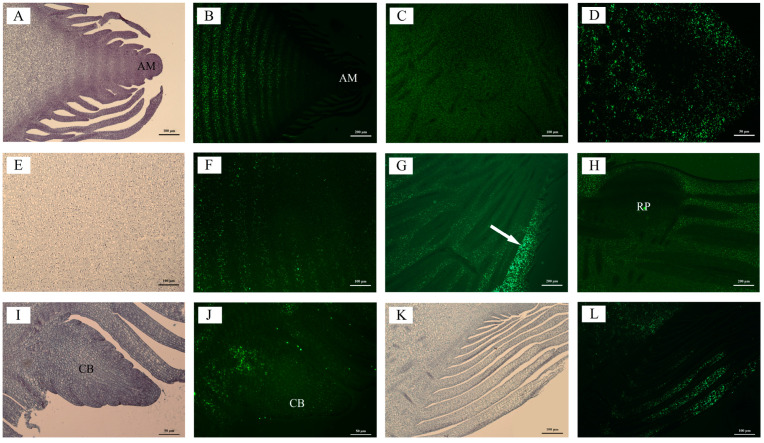
Starch granule distribution in parenchyma cells at different developmental periods of shoots, root primordium, and culm buds. (**A**) Longitudinal section of bamboo shoot bud at the late developmental stage to show the morphological characteristics of the apical meristem; (**B**) fluorescence corresponding to (**A**); (**C**) fluorescence of pith meristem of buds at the dormant stage; (**D**) fluorescence of pith of buds at the germination stage; (**E**) optical anatomical structure of differentiated nodes and internodes at the middle developmental stage; (**F**) fluorescence corresponding to (**E**); (**G**) fluorescence of basic tissue on the lateral side of buds at the early developmental stage; (**H**) fluorescence of root primordium of shoots at the mature stage; (**I**) anatomical structure of culm buds at the mature stage; (**J**) fluorescence corresponding to (**I**); (**K**) anatomical structure of bamboo shoot sheaths at the germination stage; and (**L**) fluorescence corresponding to (**K**). AM, apical meristem; RP, root primordium; CB, culm bud; The white arrow indicates the presence of starch granules within the fundamental parenchyma.

Starch granules increased with the development of shoot buds, consistent with changes in starch content (Figure 3, Figures S1 and S2). The distribution of starch granules exhibited a gradual decline from the base to the upper portion of the buds during the initial stages of development (Figure 3(A1–B3), Figures S1 and S2(A1–D2)). Subsequently, as the root primordium underwent differentiation at the late developmental stage, a decrease in the number of starch granules was observed at the base in comparison to the middle and upper sections. This trend persisted until the mature stage (Figure 3(C1–C3) and Figure S2(E1–L2)). The apical meristem exhibited a lack of starch granules throughout the shoot bud development process (Figure 4A,B, Figures S1,D2,H1,H2,L1,L2) and S2(D1,D2,H1,H2,L1,L2)). Starch granules accumulated in the pith at the germination stage and gradually increased with the development of buds (Figure 4E,F, Figures S1(C1,C2,G1,G2,K1,K2) and S2(C1,C2,G1,G2,K1,K2)). A clear difference in the distribution of starch granules between nodes and internodes was observed from the middle developmental stage (Figure 4E,F). The parenchyma cells of the basic tissue, particularly surrounding the root primordium, exhibited a significant abundance of starch granules (Figure 4G,H). Starch granules in culm buds were mainly distributed at the base (Figure 4I,J). Notably, starch granules were also found in bamboo sheaths, except the upper bamboo sheaths, which were at the early stage of development (Figure 4K,L).

**Figure 5:**
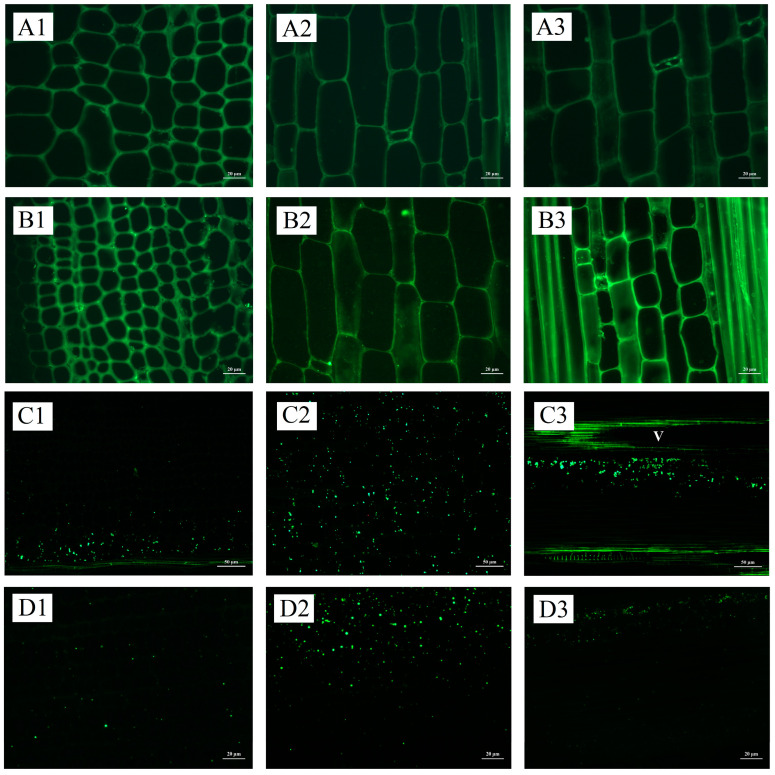
Starch granule distribution in different internodes of spring bamboo shoots. (**A1**) The inner part of the base internode; (**A2**) the middle part of the base internode; (**A3**) the outer part of the base internode; (**B1**) the inner part of the middle internode; (**B2**) the middle part of the middle internode; (**B3**) the outer part of the middle internode; (**C1**) the inner part of the upper internode; (**C2**) the middle part of the upper internode; (**C3**) the outer part of the upper internode; (**D1**) the inner part of the top internode; (**D2**) the middle part of the top internode; and (**D3**) the outer part of the top internode. V, vessel.

There were few starch granules at the base and middle of the spring bamboo shoots, with more starch granules in the upper and top internodes, but starch granules in the top internodes were still significantly degraded compared to winter shoots (Figure 5). Starch granules were more abundant in the middle and outer parts of internodes when there were fewer granules in the inner part. In addition, the distribution of granules was more concentrated around the vascular bundles (Figure 5(C3)).

### 2.2. Changes in Non-Structural Carbon Hydration of Culms

**Figure 6:**
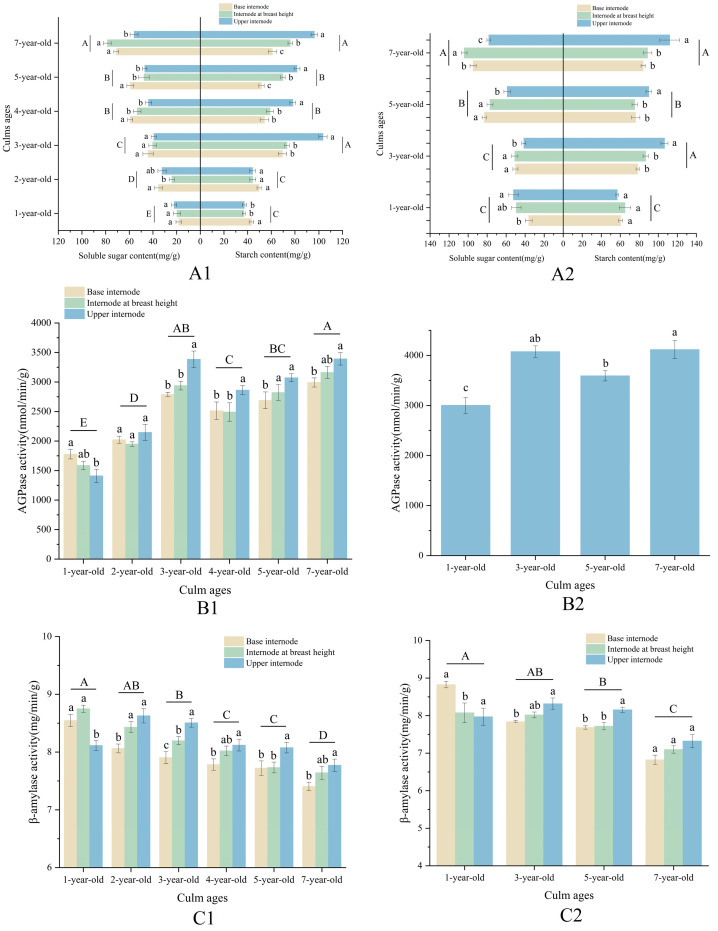
Changes in NSC contents and starch-metabolising enzyme activities in different parts of the bamboo culms at different ages in winter and spring. (**A1**) Contents of NSCs in different parts of the bamboo culms at different ages in winter; (**A2**) contents of NSCs in different parts of the bamboo culms at different ages in spring; (**B1**) AGPase activities in different parts of the bamboo culms at different ages in winter; (**B2**) AGPase activities in internode at breast height of the bamboo culms at different ages in spring; (**C1**) β-amylase activities in different parts of the bamboo culms at different ages in winter; and (**C2**) β-amylase activities in different parts of the bamboo culms at different ages in spring. Different letters in each graph indicate significant differences, *p* < 0.05.

The contents of starch, soluble sugar, and activities of AGPase and β-amylase were significantly different in bamboo culms of different ages (Figure 6). Generally, as culm age increased, soluble sugar content increased, β-amylase activity decreased, while both starch content and AGPase activity showed an N shape with the turning points observed in culms that were 3 and 4 years old. Starch content and AGPase activity were higher in 3-year-old culms compared to culms of other ages, except for those that were 7 years old. On the other hand, 1-year-old and 2-year-old culms had lower levels of starch content and AGPase activity. Starch content, soluble sugar content, and β-amylase activity were higher, and AGPase activity was lower in spring, although the trends were similar to those in winter.

The ages of bamboo culms had an impact on the starch, soluble sugar contents, and starch-metabolising enzyme activities in different internodes (Figure 6). Specifically, bamboo culms aged three years and older consistently exhibited decreased soluble sugar content and β-amylase activity, as well as increased starch content from the base internodes to the upper internodes, except for 7-year-old culms, where the highest content of soluble sugars was found in the internodes at breast height. These differences were generally significant across the different internodes of the culms. Conversely, 1-year-old bamboo culms displayed a lower content of soluble sugars in the base internodes and internodes at breast height while exhibiting higher levels of starch content and enzyme activity. Changes in the indicators in 2-year-old bamboo culms differed from those observed in culms of other ages.

**Figure 7:**
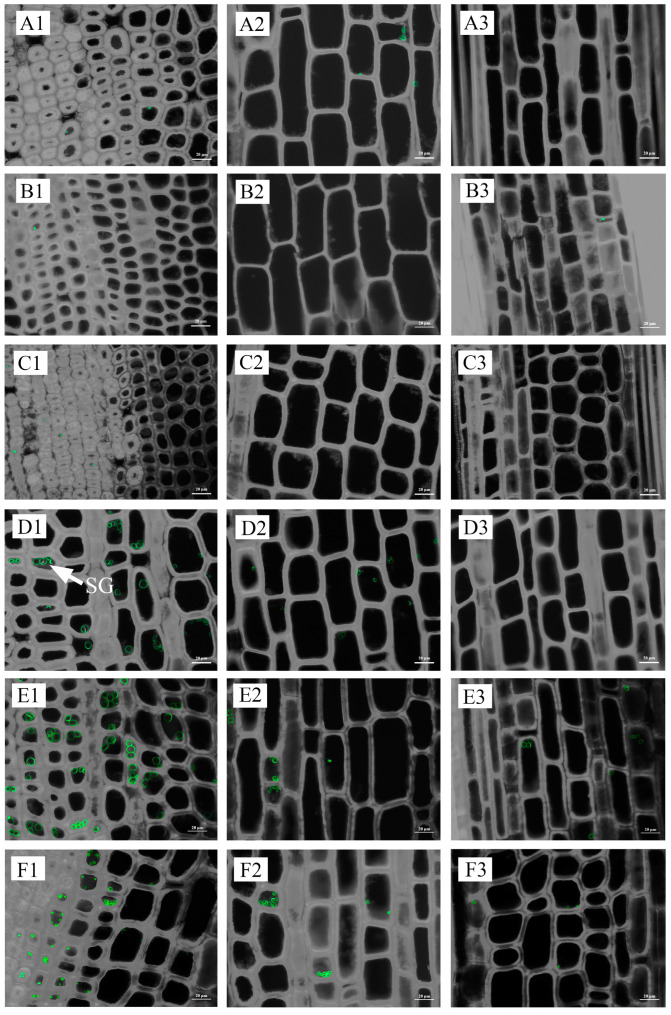
Distribution of starch granules in 1-year-old and 5-year-old bamboo culms in winter. (**A1**) The inner part of the base internode of a 1-year-old culm; (**A2**) the middle part of the base internode of a 1-year-old culm; (**A3**) the outer part of the base internode of a 1-year-old culm; (**B1**) the inner part of the internode at breast height of a 1-year-old culm; (**B2**) the middle part of the internode at breast height of a 1-year-old culm; (**B3**) the outer part of the internode at breast height of a 1-year-old culm; (**C1**) the inner part of the upper internode of a 1-year-old culm; (**C2**) the middle part of the base internode of a 1-year-old culm; (**C3**) the outer part of the base internode of a 1-year-old culm; (**D1**) the inner part of the base internode of a 5-year-old culm; (**D2**) the middle part of the base internode of a 5-year-old culm; (**D3**) the outer part of the base internode of a 5-year-old culm; (**E1**) the inner part of the internode at breast height of a 5-year-old culm; (**E2**) the middle part of the internode at breast height of a 5-year-old culm; (**E3**) the outer part of the internode at breast height of a 5-year-old culm; (**F1**) the inner part of the upper internode of a 5-year-old culm; (**F2**) the middle part of the base internode of a 5-year-old culm; and (**F3**) the outer part of the base internode of a 5-year-old culm. SG, starch granules.

**Figure 8:**
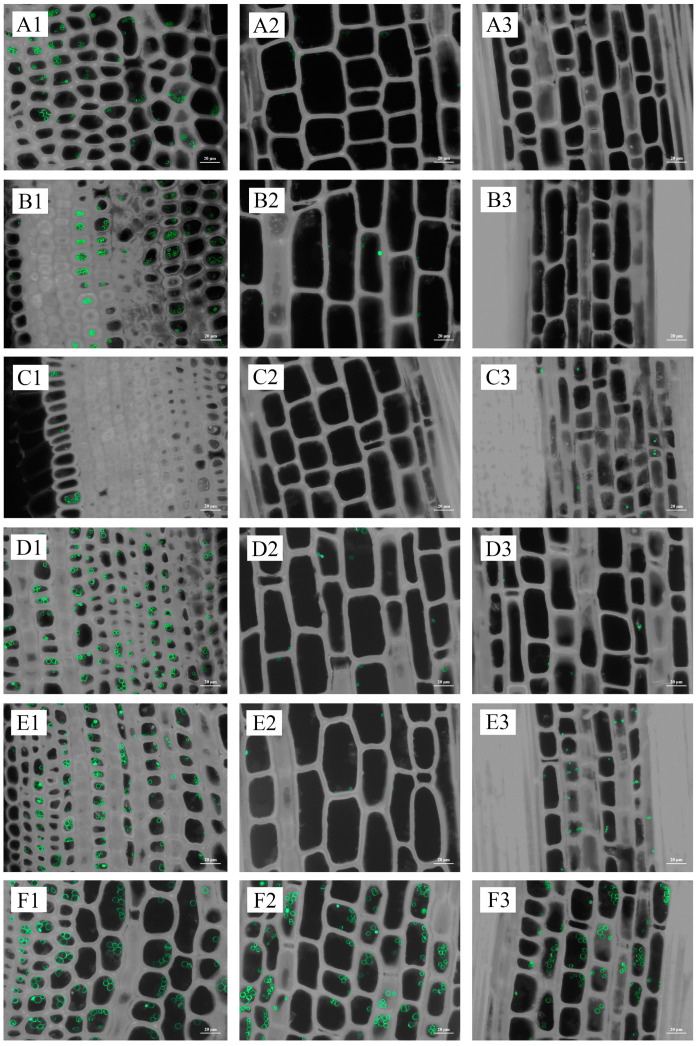
Distribution of starch granules in 1-year-old and 5-year-old bamboo culms in spring. (**A1**) The inner part of the base internode of a 1-year-old culm; (**A2**) the middle part of the base internode of a 1-year-old culm; (**A3**) the outer part of the base internode of a 1-year-old culm; (**B1**) the inner part of the internode at breast height of a 1-year-old culm; (**B2**) the middle part of the internode at breast height of a 1-year-old culm; (**B3**) the outer part of the internode at breast height of a 1-year-old culm; (**C1**) the inner part of the upper internode of a 1-year-old culm; (**C2**) the middle part of the base internode of a 1-year-old culm; (**C3**) the outer part of the base internode of a 1-year-old culm; (**D1**) the inner part of the base internode of a 5-year-old culm; (**D2**) the middle part of the base internode of a 5-year-old culm; (**D3**) the outer part of the base internode of a 5-year-old culm; (**E1**) the inner part of the internode at breast height of a 5-year-old culm; (**E2**) the middle part of the internode at breast height of a 5-year-old culm; (**E3**) the outer part of the internode at breast height of a 5-year-old culm; (**F1**) the inner part of the upper internode of a 5-year-old culm; (**F2**) the middle part of the base internode of a 5-year-old culm; and (**F3**) the outer part of the base internode of a 5-year-old culm.

The quantity of starch granules and starch content generally followed the same trend. For an internode, starch granules were concentrated on the inner side (Figure 7 and Figure 8; Figures S3–S7).

### 2.3. Changes in Non-Structural Carbon Hydration of Rhizomes

**Figure 9:**
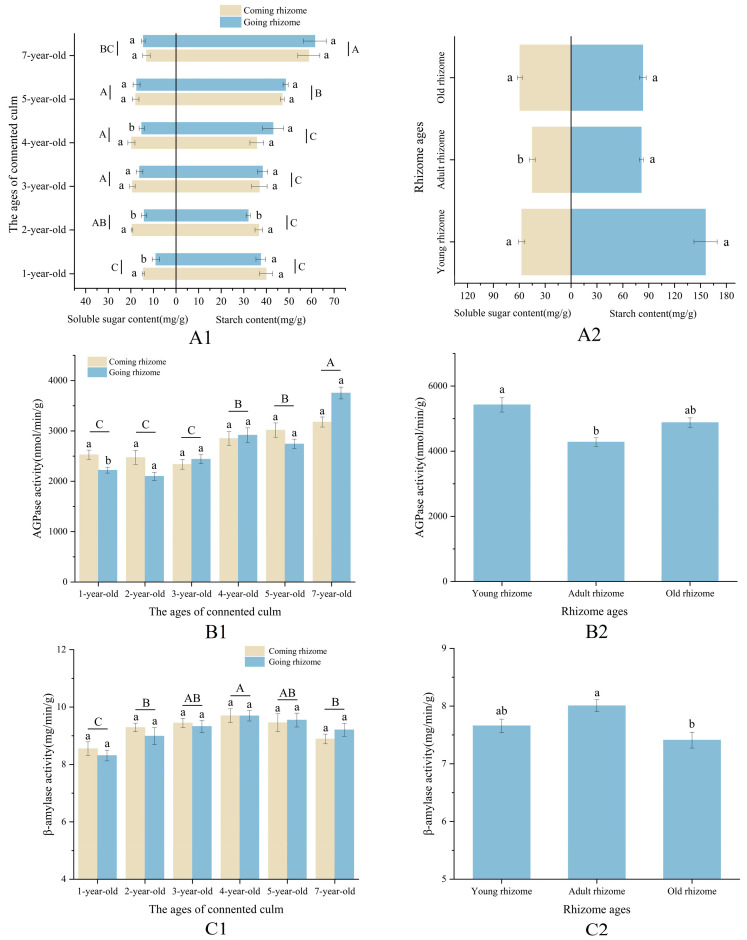
Changes in NSC content and starch-metabolising enzyme activities in rhizomes. (**A1**) NSC contents in coming rhizomes and going rhizomes connected with bamboo culms at different ages in winter; (**A2**) contents of NSCs in rhizomes of different ages in spring; (**B1**) AGPase activity in coming rhizomes and going rhizomes connected with bamboo culms at different ages in winter; (**B2**) AGPase activity in rhizomes at different ages in spring; (**C1**) β-amylase activity in coming rhizomes and going rhizomes connected with bamboo culms at different ages in winter; (**C2**) β-amylase activity in rhizomes at different ages in spring. Different letters in each graph indicate significant differences, *p* < 0.05.

Soluble sugars, starch content, and AGPase and β-amylase activities in rhizomes were affected not only by the age of rhizomes but also by the age of connected culms (Figure 9). In general, there was a significant increase in starch and soluble sugar contents, as well as AGPase activity, while β-amylase activity exhibited a significant decrease from winter to spring. Notably, the starch content, AGPase, and β-amylase activities displayed the same trends during winter and spring, whereas the soluble sugar content in rhizomes exhibited contrasting patterns.

Specifically, as the age of the connected culms increased, there was a decrease and subsequent increase in starch content and AGPase activity in the rhizomes, both in spring and winter, resembling a V shape. Conversely, the trend of β-amylase activity exhibited an inverted V shape, with the turning point occurring at the adult rhizomes. The soluble sugar content in the rhizomes displayed variations across different seasons, characterised by an inverted V shape during winter and a V shape during spring, attributed to the slower rate of increase in soluble sugar content in the adult rhizomes.

**Figure 10:**
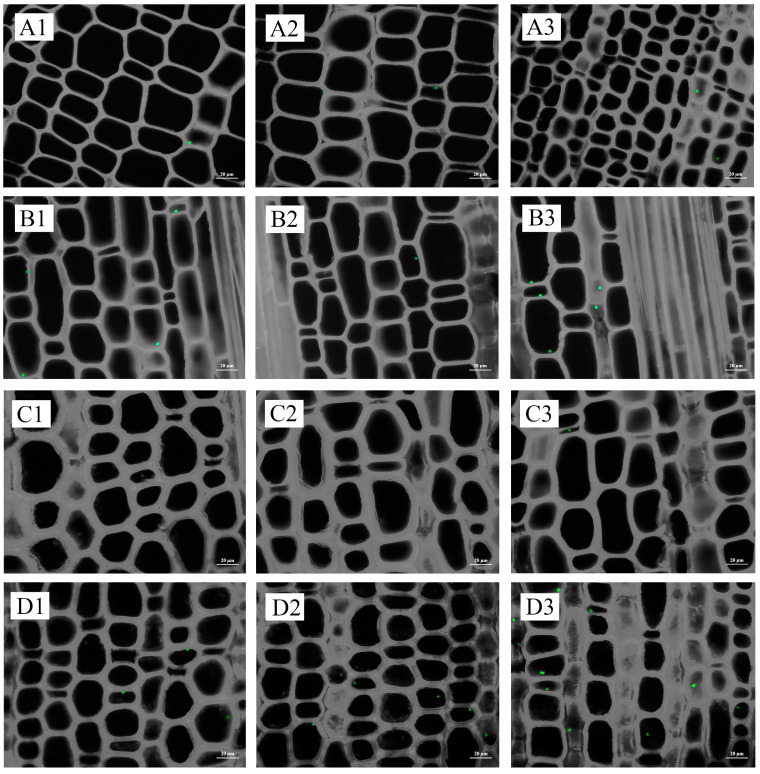
Distribution of starch granules in the rhizome connected with 1-year-old and 5-year-old bamboo culms in winter. (**A1**) The inner part of the coming rhizome connected with a 1-year-old culm; (**A2**) the middle part of the coming rhizome connected with a 1-year-old culm; (**A3**) the outer part of the coming rhizome connected with a 1-year-old culm; (**B1**) the inner part of the going rhizome connected with a 1-year-old culm; (**B2**) the middle part of the going rhizome connected with a 1-year-old culm; (**B3**) the outer part of the going rhizome connected with a 1-year-old culm; (**C1**) the inner part of the coming rhizome connected with a 5-year-old culm; (**C2**) the middle part of the coming rhizome connected with a 5-year-old culm; (**C3**) the outer part of the coming rhizome connected with a 5-year-old culm; (**D1**) the inner part of the going rhizome connected with a 5-year-old culm; (**D2**) the middle part of the going rhizome connected with a 5-year-old culm; and (**D3**) the outer part of the going rhizome connected with a 5-year-old culm.

**Figure 11:**
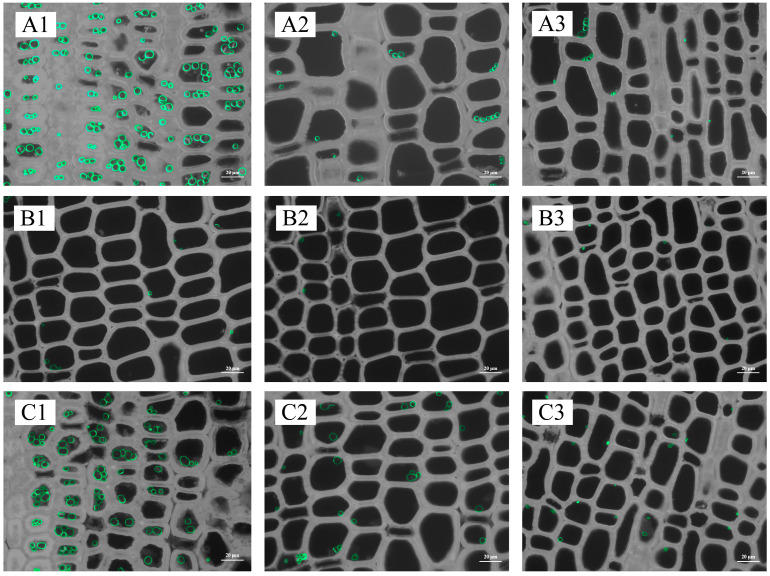
Distribution of starch granules in rhizome in spring. (**A1**) The inner part of the young rhizome; (**A2**) the middle part of the young rhizome; (**A3**) the outer part of the young rhizome; (**B1**) the inner part of the adult rhizome; (**B2**) the middle part of the adult rhizome; (**B3**) the outer part of the adult rhizome; (**C1**) the inner part of the old rhizome; (**C2**) the middle part of the old rhizome; and (**C3**) the outer part of the old rhizome.

Hence, adult rhizomes exhibited lower levels of starch content and AGPase activity while displaying higher levels of β-amylase activity and soluble sugar content in the winter and lower levels in the spring when compared to old and young rhizomes. However, the variations in starch content, soluble sugar content, AGPase activity, and β-amylase activity between coming and going rhizomes of interconnected culms were generally found to be statistically insignificant across most stages of development. Furthermore, the distribution of starch granules across different seasons and ages exhibited a similar pattern to the changes observed in starch content. Specifically, winter rhizomes displayed a scarcity of starch granules, whereas spring rhizomes, particularly in the inner regions of young rhizomes, exhibited a substantial abundance of starch granules (Figure 10 and Figure 11; Figures S8 and S9).

## 3. Discussion

### 3.1. Metabolism of NSCs Preceded the Development of Bamboo Shoots

Carbon in bamboo shoots originated from the parent bamboo and was closely related to the development of bamboo shoots. The accumulation or consumption of NSCs in bamboo shoots depends on the carbon “supply and demand”. During winter, the slow growth of shoots leads to the manifestation of starch granule accumulation. Conversely, in spring, the rapid growth of shoots results in the degradation of starch granules due to accelerated sugar utilisation despite the efficient transport of sugar from the parent bamboo [44]. The order of starch accumulation and consumption in the longitudinal direction of the shoot was consistent with its developmental sequence, progressing sequentially from the base internode of the bamboo shoot [45]. Our previous study on starch granules of *Ph. edulis* ‘Pachyloen’ shoots during elongation growth agrees with the results [46].

It is noteworthy that the accumulation and consumption of starch granules appear to occur slightly prior to the differentiation of the tissues. The clear evidence was that starch granules accumulated in large quantities before the rapid proliferation and elongation of the cells. Starch also aggregated earlier around the root primordia and at the base of culm buds before they developed. Moreover, the starch granules in the top internodes, which were in the elongation phase in spring, were already heavily degraded; this may be related to the property of starch granules to store NSCs for future use [47].

The functions of bamboo sheaths have received increasing attention in recent years. Some researchers believe that sheaths not only play the role of protecting, supporting, and providing a suitable environment for the growth of bamboo shoots but also participate in water, carbon metabolism, and even photosynthesis [6,7,8]. Bamboo sheaths had complete photosynthetic system genes, and the photosynthetic capacity of sheath blades was improved with their development. Ding et al. suggested that bamboo sheaths and shoots might be a source–sink relationship [48]. In this study, we found that sheaths had the ability to store starch. Since underground shoot buds were unable to photosynthesise, NSCs were transported from the bud itself through the vascular bundles to the sheaths and stored in them [7,49]. Some studies have shown that starch granules were also present in the sheaths during the elongation growth [6], and the starch granules at this time may originate from their own photosynthesis or may be transported to the sheaths together with water from parent bamboo, which is still a mystery and still needs to be further researched.

### 3.2. Bamboo Culms of Different Ages Have Different Functions during the Development of Bamboo Shoots

The supply of carbon to bamboo shoots exhibited variability across various ages of parent bamboo, depending on the availability of carbon reserves and physiological conditions. In general, the physiological metabolisms of young and adult bamboo culms were vigorous, and those of old culms were slow. It is widely acknowledged that bamboo culms do not exhibit increased height or thickness following the first growing season, but the deposition and lignification of the various types of cell walls, such as fibre cells and parenchyma cells, in bamboo culms continues. The studies demonstrated that the process of secondary wall thickening exhibited the highest rate in young culms, experienced a deceleration in adult culms, and displayed a significantly reduced pace in old culms [50,51,52]. The sap flow rate showed the same pattern [53,54]. Photosynthesis was enhanced and then reduced as the age of the bamboo culms increased. Cao et al. found that the photosynthetic rate of leaves in the middle layer of moso bamboo was 3-year-old > 4-year-old > 2-year-old > 7-year-old culms under different light intensities [55]. Photosynthesis in old bamboo culms was three times slower than in young culms [56]. The income and expenditure relationship of carbon resulted in the lowest carbon reserves in young culms and higher carbon reserves in adult and old culms, especially the starch storage capacity of 3-year-old culms, which was stronger compared to other ages of adult culms and had the material basis for supplying NSCs to shoots. According to the enzyme activities, lower AGPase activity and higher β-amylase activity of adult culms were more beneficial for the transport of soluble sugars, while higher AGPase activity and lower β-amylase activity of old bamboo culms were more suitable for starch storage, which reflected the differences in function of culms in different ages. Moreover, it was proved that the vascular bundles in the very old culms were clogged, resulting in the blockage of substance transport [54,57]. Obviously, the adult culms have more favourable conditions to provide NSCs for bamboo shoots. Due to the water–carbon collaborative transport, the direction of water flow from high to low water potential in the bamboo stand determined the direction of carbon transport [19,53]. During this process, bamboo culms of different ages contributed carbon to the shoots, but we believe that the adult culm contributed more carbon to the system, especially the 3-year-old culm; this might also be influenced by the standing conditions and bamboo stand structure.

Old culms, which contributed less to bamboo shoots, and their timber properties began to deteriorate above 8 years old, were recommended to be prioritised for felling [58]. In addition, this study also has theoretical significance for water and fertiliser management and bamboo stand structure adjustment.

### 3.3. Supply Patterns of NSCs in Bamboo Rhizome–Culm System

Divergent perspectives among researchers exist regarding the role of rhizomes, with certain scholars positing that the primary function of rhizomes lies in the transportation of sugars, while others contending that rhizomes additionally serve as reservoirs for starch granules [40,43]. Our research has found that rhizomes at different ages exhibited different functions. Young and old rhizomes exhibited higher levels of starch content and AGPase activity, whereas adult rhizomes displayed elevated β-amylase activity. This study provides evidence demonstrating the correlation between the age of rhizomes and the age of connecting bamboo culms with regard to their respective functions. Therefore, it was plausible that adult rhizomes exhibited a greater propensity for facilitating the transportation of soluble sugars, while young and old rhizomes had an increased function to store starch granules and a reduced transport of soluble sugars with fewer shoot buds sprouting [59]. The bamboo shoot buds on young rhizomes of the current year were usually dormant. In contrast, adult rhizomes exhibited heightened dynamism, with their roots displaying superior absorptive capabilities and their lateral buds undergoing germination [60]. The old rhizomes and their lateral buds undergo a process of darkening or decay, with the loss of most rhizome roots and their diminished capacity for nutrient absorption [61]. However, studies on the physiological metabolism and developmental mechanisms of rhizomes of different ages were very scarce, and this paper only revealed their functional differences at the level of carbon supply. More in-depth studies at other levels are still needed.

**Figure 12:**
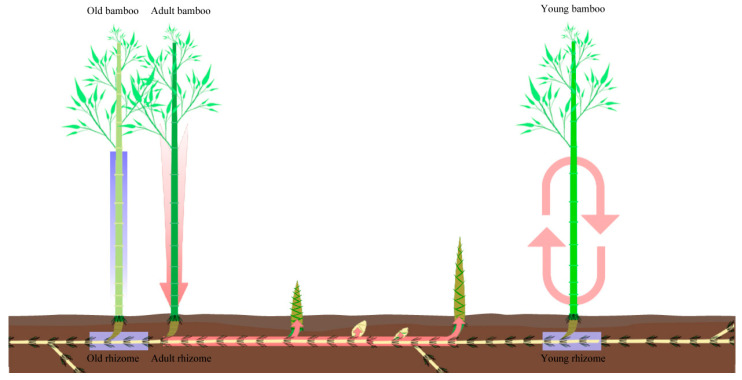
NSC supply pattern. Blue stripes indicate starch storage and pink stripes indicate soluble sugar transport. The arrow points in the direction of soluble sugar transport.

Based on the above results, a possible NSC supply pattern was speculated (Figure 12). In winter, more sugars were transported downward to the rhizomes from the adult culms than from the young and old culms. Similarly, when starch was stored in young and old rhizomes, more starch was converted to soluble sugars in adult rhizomes for transport to the shoot buds and storage at the site where it would develop. The pattern of NSCs supply in spring was similar to that in winter, but more soluble sugars flowed from the culms into the rhizomes and reached the bamboo shoots. Starch stored in shoots was also converted earlier to soluble sugars due to rapid growth. The direction of NSCs flow in the rhizome–culm system during this process is not clear. Although there was a pattern of cooperative transport between water and carbon in the rhizomes, some researchers believed that the water potential was the highest in the old bamboo culms during the development of shoot [62], and others believed that the water potential was higher in the adult culms [63]. We will use isotope tracer methods to explore the pathways of material flow. In addition, the mechanism of substance metabolism in bamboo shoots during the rapid elongation growth still needs further study.

## 4. Materials and Methods

### 4.1. Plant Materials

**Figure 13:**
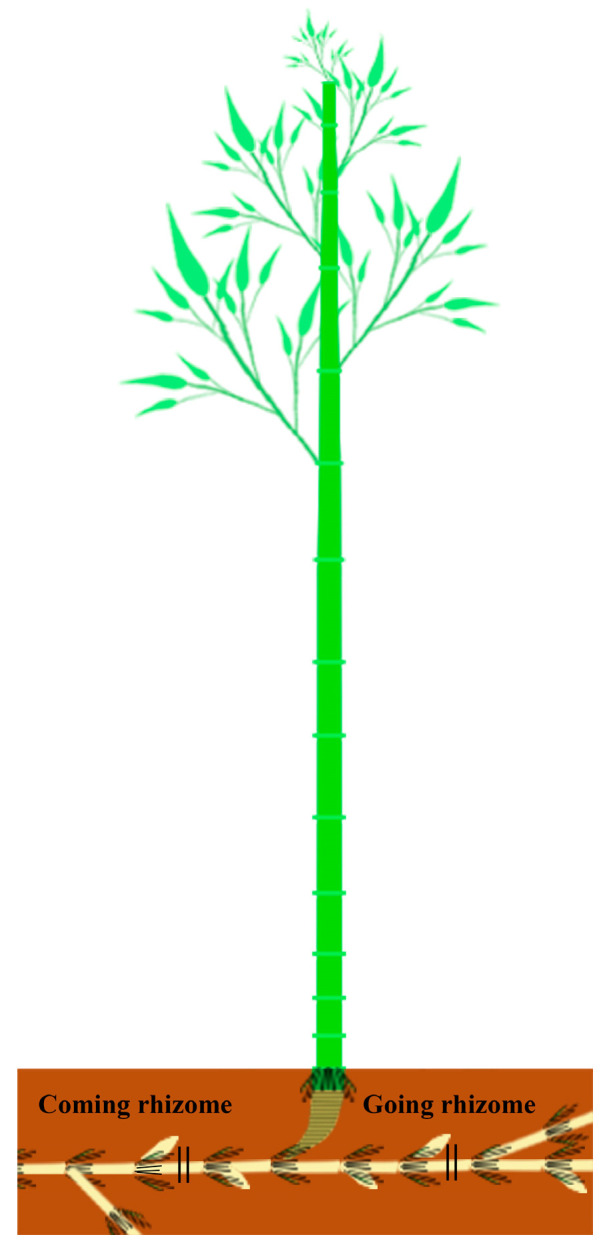
Schematic diagram of the rhizome sampling site. || indicates cutting sites of the internodes of the coming and going rhizomes.

*Ph. edulis* materials were collected from Cheshang Village Forestry Centre (28°25′12″ N, 114°33′36″ E) in Yifeng County, Yichun City, Jiangxi Province, and the Bamboo Garden (28°46′30″ N, 115°49′23″ E) of Jiangxi Agricultural University. Both places have a subtropical monsoon climate. The mean annual temperatures at Cheshang Village Forestry Centre and the Bamboo Garden of Jiangxi Agricultural University were 19.61 °C and 19.58 °C, respectively, while the precipitation was 1486.28 mm and 1567.94 mm. The soil types at both sites were red loam. Bamboo stands are similar in condition and structure. Bamboo shoots, culms, and rhizomes were harvested during the primary thickening growth of shoot buds in winter 2021 and elongation growth in spring 2022. Shoot buds from each stage, the middle part of the base internodes, the internodes at breast height, and the upper internodes of bamboo culms aged 1 to 5 and 7 years in winter and bamboo culms aged 1, 3, 5, and 7 years in spring were collected. It is worth noting that culms at the age of 1 year were considered young, while culms aged 2 to 5 years were classified as adult, and culms aged 7 years were considered old. Furthermore, the middle parts of the internodes of both coming and going rhizomes in winter (Figure 13), along with the middle sections of the internodes from young, adult, and old rhizomes, categorised based on morphological characteristics in spring, were also harvested. At least three healthy samples of each material in a similar state of growth and without damage were taken.

### 4.2. Observation of Cell Morphology and Starch Granules

Samples of the bud and bamboo shoot were cut into small pieces that were approximately 5 mm × 5 mm × 10 mm and fixed in 50% FAA. The culms and rhizomes were cut into small pieces approximately 5 mm × 5 mm × thick and fixed with 70% FAA. All fixed samples were exhausted with a vacuum pump. Samples with low lignification were used in paraffin sections, and samples with high lignification were used in polyethylene glycol (PEG: 2000) sections with a thickness of 8 μm.

The slices were observed and photographed under a light microscope and a fluorescence microscope (Zeiss AXIO, Oberkochen, Germany) with an excitation light source (bandwidth 470/40 nm; emission wavelength 525/50 nm). The fluorescence of the cell wall in culms and rhizomes was removed, leaving only the fluorescence emitted by starch granules.

### 4.3. Determination of Soluble Sugar and Starch Contents

Following their drying and crushing, 0.1 g samples were added to 10 mL of 80% alcohol, heated in a water bath at 80 °C for 30 min, and filtered; this process was repeated three times. The liquid was used for the determination of soluble sugar content. Starch was extracted three times by adding 5 mL of dilute acid to the precipitate, followed by water-bath heating at 80 °C for 30 min and filtration. The filtrate was used for the determination of starch content. The anthrone colourimetric method was employed to determine the soluble sugar content and starch content of the samples. In total, 4 mL of anthrone solution was added to the diluted filtrate and heated at 100 °C for 15 min. The absorbance at 620 nm was used to determine the soluble sugar and starch contents.

### 4.4. Starch-Related Enzyme Activity Measurement

The samples were cut into small pieces, wrapped tightly with tin foil, labelled and put into liquid nitrogen for rapid freezing, and stored at −80 °C.

AGPase and β-amylase activities were measured using commercially available kits (Suzhou Comin Biotechnology Co., Ltd., Suzhou, China).

### 4.5. Statistical Analyses

SPSS 22.0 was used for data analysis. One-way ANOVA was used to compare differences in starch, soluble sugars, and starch-metabolising enzyme activities, and multiple comparisons were made using the least significant difference method (LSD), and the differences were considered significant when *p* < 0.05. Plots were made using OriginPro 2022.

## 5. Conclusions

NSCs were integrated and deployed in the rhizome–culm system during bamboo shoot development. Among the bamboo culms and rhizomes of different ages, the adult culm and adult rhizome were more suitable for the transport of NSCs and might have the potential to supply more NSCs to shoots. In contrast, young culms seemed to prefer their growth, while old culms and young and old rhizomes tended to store NSCs, which were related to their growth conditions and physiological characteristics. NSCs arriving in bamboo shoots were stored in winter and consumed in spring, and the timing of their accumulation and consumption coincided with and preceded the process of development. In addition, bamboo sheaths had the function of starch storage and participated in the metabolism of NSCs in bamboo shoots. Our findings establish the groundwork for future investigations into the growth of bamboo shoots and the underlying mechanisms of substance metabolism within bamboo stands. Additionally, this study offers valuable insights for optimising the structure of bamboo stands and implementing effective management strategies.

## Data Availability

Data supporting the results of this study are available from the authors.

## Supplementary Materials

The following supporting information can be downloaded at https://www.mdpi.com/article/10.3390/plants13010002/s1. Figure S1. Anatomical structure and starch granules distribution of winter shoot buds during the dormant stage, germination stage, and early developmental stage; Figure S2. Anatomical structure and starch granules distribution of winter shoot buds during the middle developmental stage, late developmental stage, and mature stage; Figure S3. Distribution of starch granules in 1–2-year-old bamboo culms in winter; Figure S4. Distribution of starch granules in 3–4-year-old bamboo culms in winter; Figure S5. Distribution of starch granules in 5-year-old and 7-year-old bamboo culms in winter; Figure S6. Distribution of starch granules in 1-year-old and 3-year-old bamboo culms in spring; Figure S7. Distribution of starch granules in 5-year-old and 7-year-old bamboo culms in spring; Figure S8. Distribution of starch granules of the rhizome connected with 1–3-year-old bamboo culms in winter; Figure S9. Distribution of starch granules of the rhizome connected with 4, 5, and 7-year-old bamboo culms in winter.
