# Supplementary material for: Emerging Insights into the Roles of the Rhizome–Culm System in Bamboo Shoot Development through Analysis of Non-Structural Carbohydrate Changes
Source: Plants (Basel). 2023 Dec 19;13(1):2. doi: 10.3390/plants13010002 (PMC10780645; doi:10.3390/plants13010002)
Supplement: Supplementary file 1 [file plants-13-00002-s001.zip › plants-2727365-supplementary.pdf]

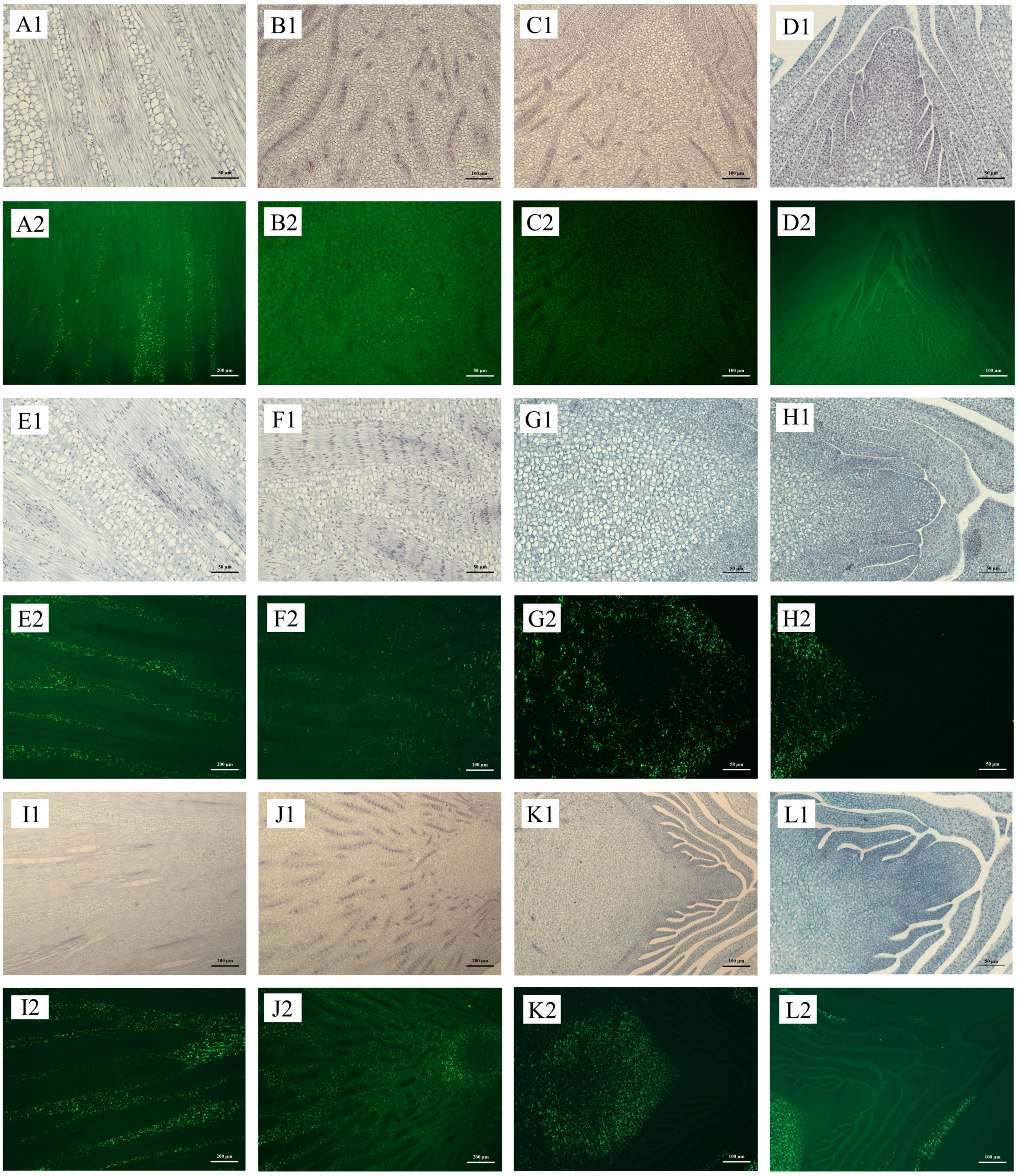

Figure S1. Anatomical structure and starch granules distribution of winter shoot buds during dormant stage, germination stage, and early developmental stage. (**A1**) Anatomical structure of the base of shoot buds at the dormant stage;(**B1**) Anatomical structure of the middle of shoot buds at the dormant stage; (**C1**) Anatomical structure of the upper part of shoot buds at the dormant stage; (**D1**) Anatomical structure of the top of shoot buds at the dormant stage; (**A2~D2**) Fluorescence corresponding to (A1~D1); (**E1**) Anatomical structure of the base of shoot buds at the germination stage; (**F1**) Anatomical structure of the middle of shoot buds at the dormant stage; (**G1**) Anatomical structure of the upper part of shoot buds at the germination stage; (**H1**) Anatomical structure of the top of shoot buds at the germination stage; (**E2~H2**) Fluorescence corresponding to (E1~H1); (**I1**) Anatomical structure of the base of shoot buds at the early developmental stage; (**J1**) Anatomical structure of the middle of shoot buds at the early developmental stage; (**K1**) Anatomical structure of the upper part of shoot buds at the early developmental stage; (**L1**) Anatomical structure of the top of shoot buds at the early developmental stage; (**I2~L2**) Fluorescence corresponding to (I1~L1).

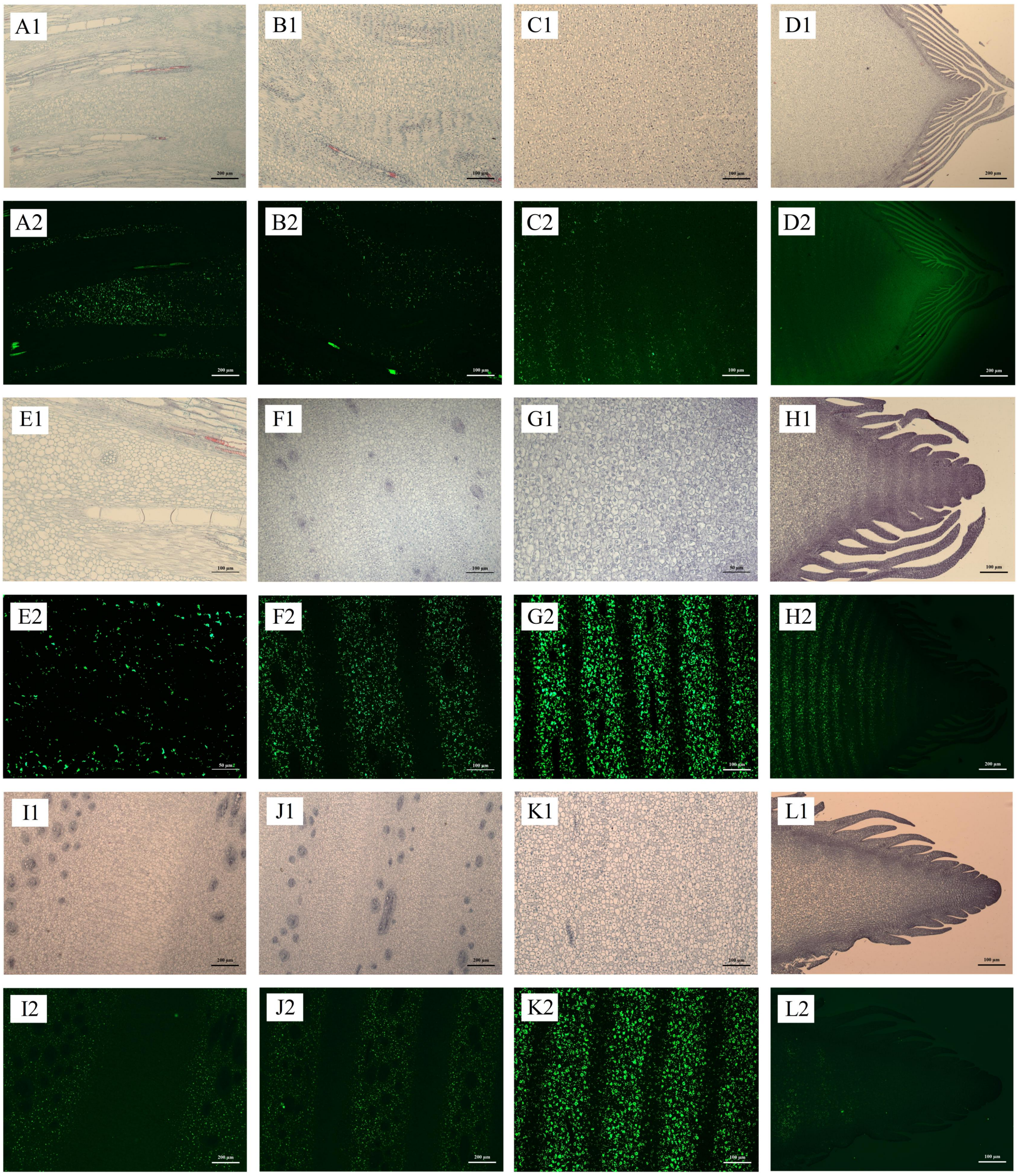

Figure S2. Anatomical structure and starch granules distribution of winter shoot buds during middle developmental stage, late developmental stage and mature stage. (**A1**): Anatomical structure of the base of shoot buds during the middle developmental stage; (**B1**) Anatomical structure of the middle of shoot buds at the middle developmental stage; (**C1**) Anatomical structure of the upper part of shoot buds at the middle developmental stage; (**D1**) Anatomical structure of the top of shoot buds at the middle developmental stage; (**A2~D2**) Fluorescence corresponding to (A1~D1); (**E1**) Anatomical structure of the base of shoot buds at the late developmental stage; (**F1**) Anatomical structure of the middle of shoot buds at the late developmental stage; (**G1**) Anatomical structure of the upper part of shoot buds at the late developmental stage; (**H1**) Anatomical structure of the top of shoot buds at the late developmental stage; (**E2~H2**) Fluorescence corresponding to (E1~H1); (**I1**) Anatomical structure of the base of shoot buds at the mature stage; (**J1**) Anatomical structure of the middle of shoot buds at the mature stage; (**K1**) Anatomical structure of the upper part of shoot buds at the mature stage; (**L1**) Anatomical structure of the top of shoot buds at the mature stage; (**I2~L2**) Fluorescence corresponding to (I1~L1).

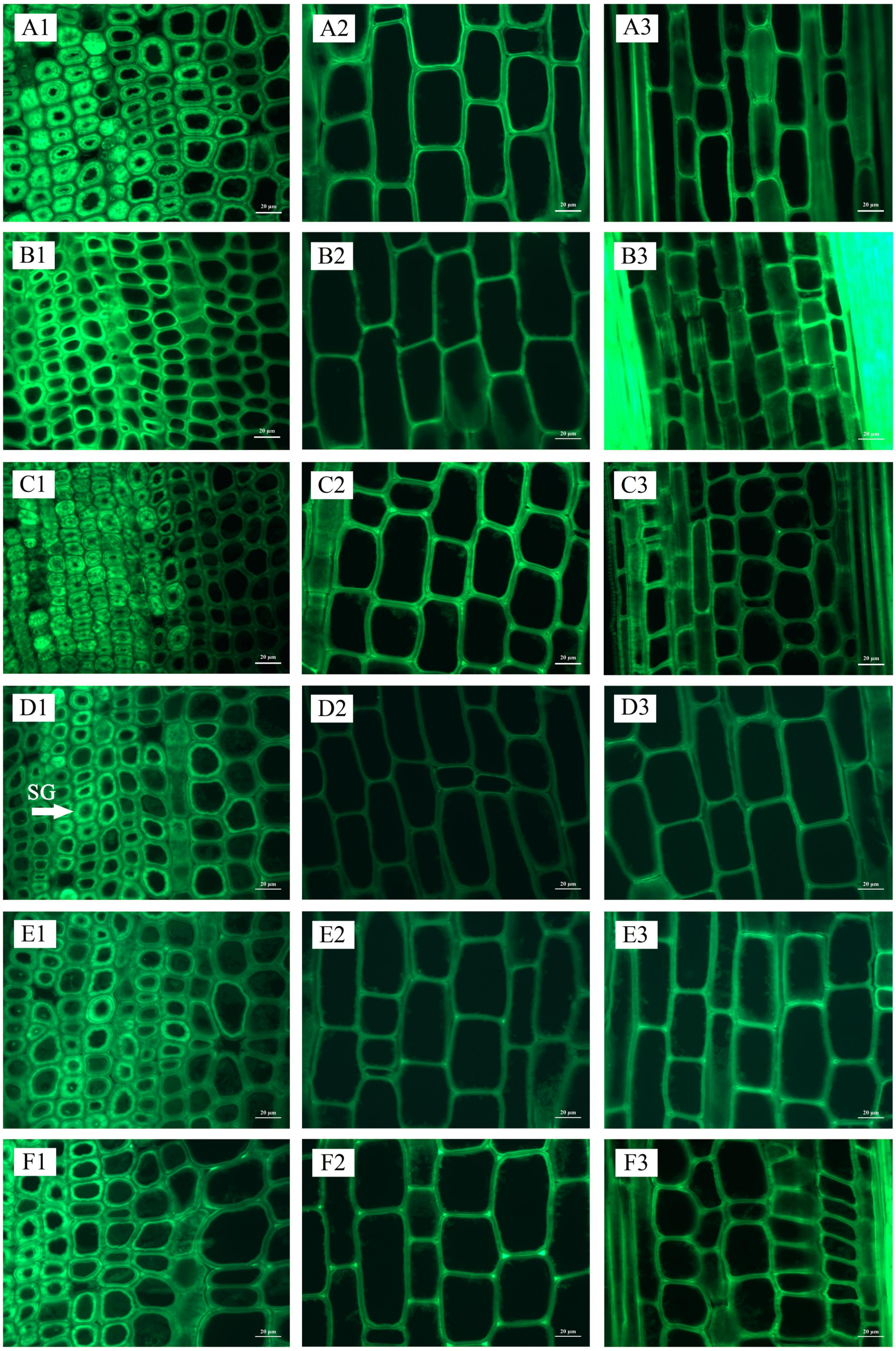

Figure S3. Distribution of starch granules in 1-2 years old bamboo culms in winter. (**A1**) The inner part of the base internode of 1-year-old culm; (**A2**) The middle part of the base internode of 1-year-old culm; (**A3**) The outer part of the base internode of 1-year-old culm; (**B1**) The inner part of the internode at breast height of 1-year-old culm; (**B2**) The middle part of the internode at breast height of 1-year-old culm; (**B3**) The outer part of the internode at breast height of 1-year-old culm; (**C1**) The inner part of the upper internode of 1-year-old culm; (**C2**) The middle part of the upper internode of 1-year-old culm; (**C3**) The outer part of the upper internode of 1-year-old culm; (**D1**) The inner part of the base internode of 2-year-old culm; (**D2**) The middle part of the base internode of 2-year-old culm; (**D3**) The outer part of the base internode of 2-year-old culm; (**E1**) The inner part of the internode at breast height of 2-year-old culm; (**E2**) The middle part of the internode at breast height of 2-year-old culm; (**E3**) The outer part of the internode at breast height of 2-year-old culm; (**F1**) The inner part of the upper internode of 2-year-old culm; (**F2**) The middle part of the upper internode of 2-year-old culm; (**F3**) The outer part of the upper internode of 2-year-old culm. SG, Starch granules.

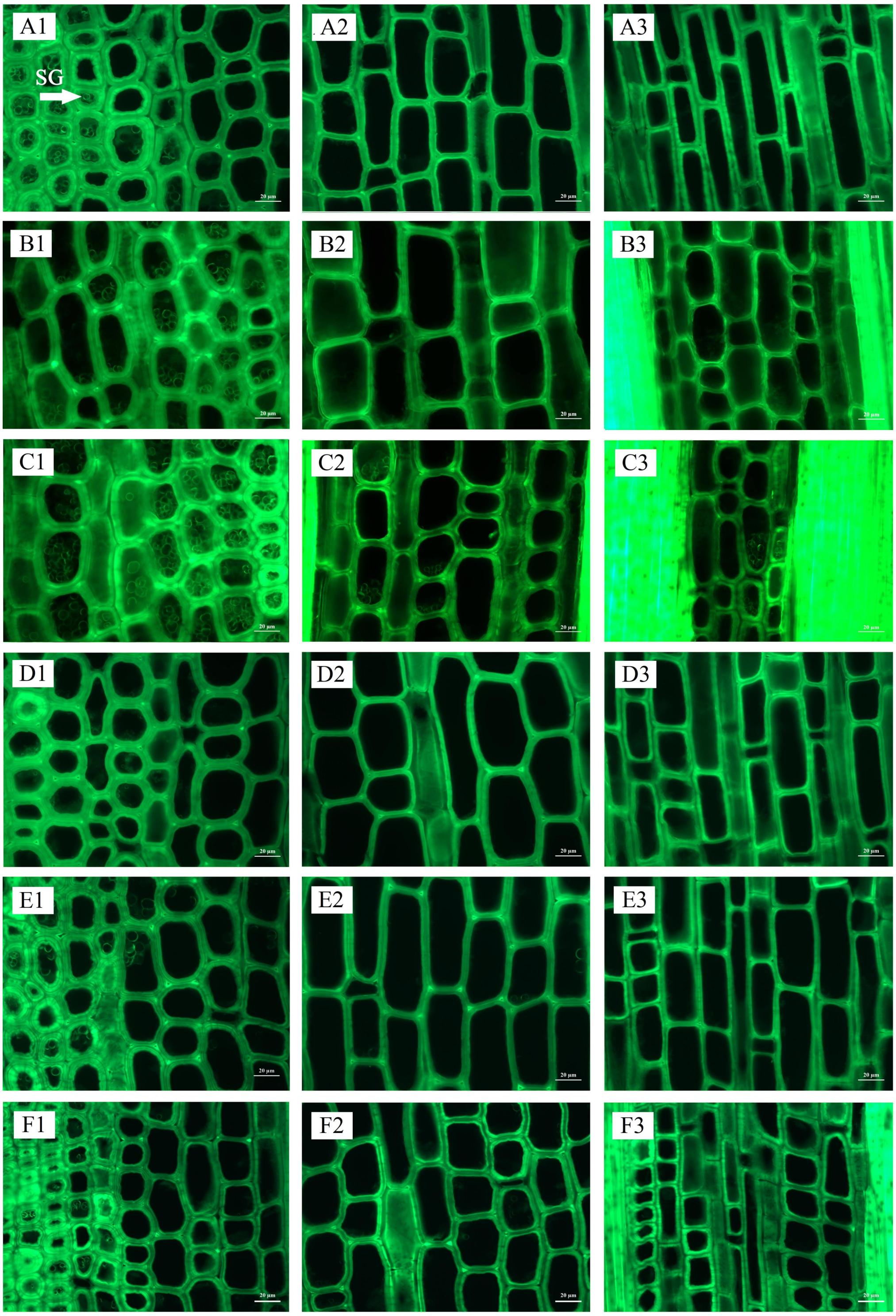

Figure S4. Distribution of starch granules in 3-4 years old bamboo culms in winter. (**A1**) The inner part of the base internode of 3-year-old culm; (**A2**) The middle part of the base internode of 3-year-old culm; (**A3**) The outer part of the base internode of 3-year-old culm; (**B1**) The inner part of the internode at breast height of 3-year-old culm; (**B2**) The middle part of the internode at breast height of 3-year-old culm; (**B3**) The outer part of the internode at breast height of 3-year-old culm; (**C1**) The inner part of the upper internode of 3-year-old culm; (**C2**) The middle part of the upper internode of 3-year-old culm; (**C3**) The outer part of the upper internode of 3-year-old culm; (**D1**) The inner part of the base internode of 4-year-old culm; (**D2**) The middle part of the base internode of 4-year-old culm; (**D3**) The outer part of the base internode of 4-year-old culm; (**E1**) The inner part of the internode at breast height of 4-year-old culm; (**E2**) The middle part of the internode at breast height of 4-year-old culm; (**E3**) The outer part of the internode at breast height of 4-year-old culm; (**F1**) The inner part of the upper internode of 4-year-old culm; (**F2**) The middle part of the upper internode of 4-year-old culm; (**F3**) The outer part of the upper internode of 4-year-old culm. SG, Starch granules.

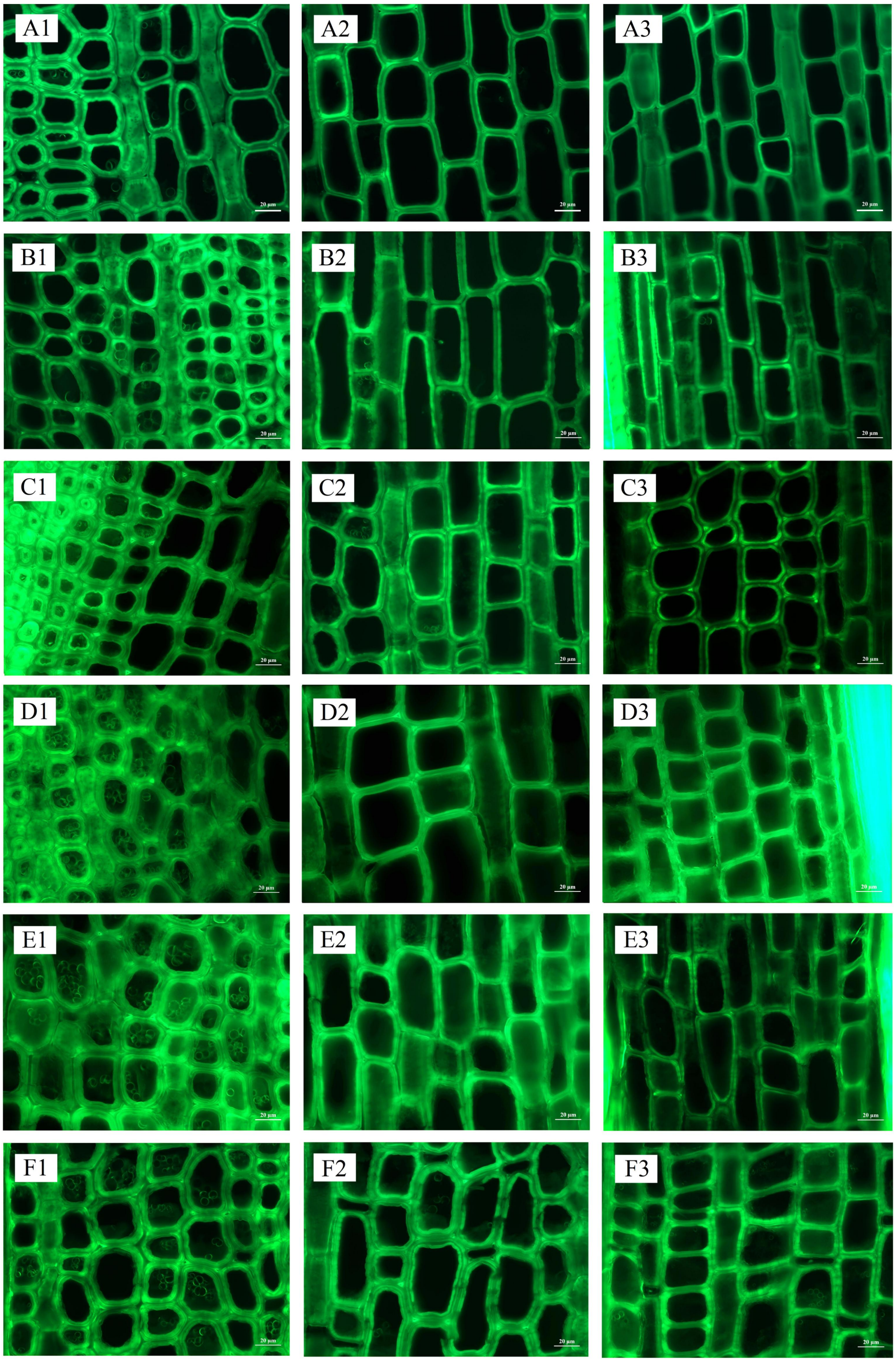

Figure S5. Distribution of starch granules in 5-year-old and 7-year-old bamboo culms in winter. (**A1**) The inner part of the base internode of 5-year-old culm; (**A2**) The middle part of the base internode of 5-year-old culm; (**A3**) The outer part of the base internode of 5-year-old culm; (**B1**) The inner part of the internode at breast height of 5-year-old culm; (**B2**) The middle part of the internode at breast height of 5-year-old culm; (**B3**) The outer part of the internode at breast height of 5-year-old culm; (**C1**) The inner part of the upper internode of 5-year-old culm; (**C2**) The middle part of the upper internode of 5-year-old culm; (**C3**) The outer part of the upper internode of 5-year-old culm; (**D1**) The inner part of the base internode of 7-year-old culm; (**D2**) The middle part of the base internode of 7-year-old culm; (**D3**) The outer part of the base internode of 7-year-old culm; (**E1**) The inner part of the internode at breast height of 7-year-old culm; (**E2**) The middle part of the internode at breast height of 7-year-old culm; (**E3**) The outer part of the internode at breast height of 7-year-old culm; (**F1**) The inner part of the upper internode of 7-year-old culm; (**F2**) The middle part of the upper internode of 7-year-old culm; (**F3**) The outer part of the upper internode of 7-year-old culm.

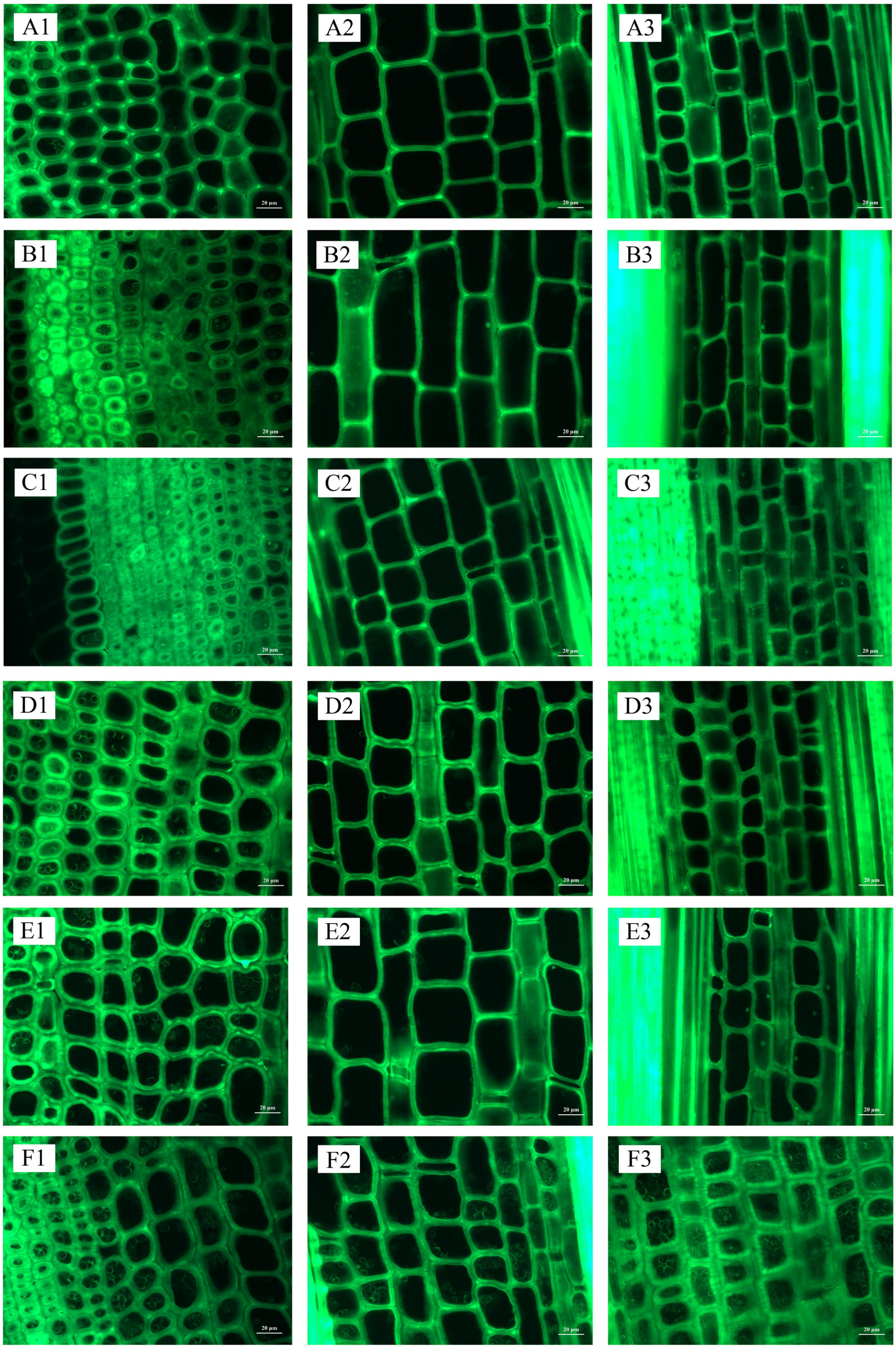

Figure S6. Distribution of starch granules in 1-year-old and 3-year-old bamboo culms in spring. (**A1**) The inner part of the base internode of 1-year-old culm; (**A2**) The middle part of the base internode of 1-year-old culm; (**A3**) The outer part of the base internode of 1-year-old culm; (**B1**) The inner part of the internode at breast height of 1-year-old culm; (**B2**) The middle part of the internode at breast height of 1-year-old culm; (**B3**) The outer part of the internode at breast height of 1-year-old culm; (**C1**) The inner part of the upper internode of 1-year-old culm; (**C2**) The middle part of the upper internode of 1-year-old culm; (**C3**) The outer part of the upper internode of 1-year-old culm; (**D1**) The inner part of the base internode of 3-year-old culm; (**D2**) The middle part of the base internode of 3-year-old culm; (**D3**) The outer part of the base internode of 3-year-old culm; (**E1**) The inner part of the internode at breast height of 3-year-old culm; (**E2**) The middle part of the internode at breast height of 3-year-old culm; (**E3**) The outer part of the internode at breast height of 3-year-old culm; (**F1**) The inner part of the upper internode of 3-year-old culm; (**F2**) The middle part of the upper internode of 3-year-old culm; (**F3**) The outer part of the upper internode of 3-year-old culm.

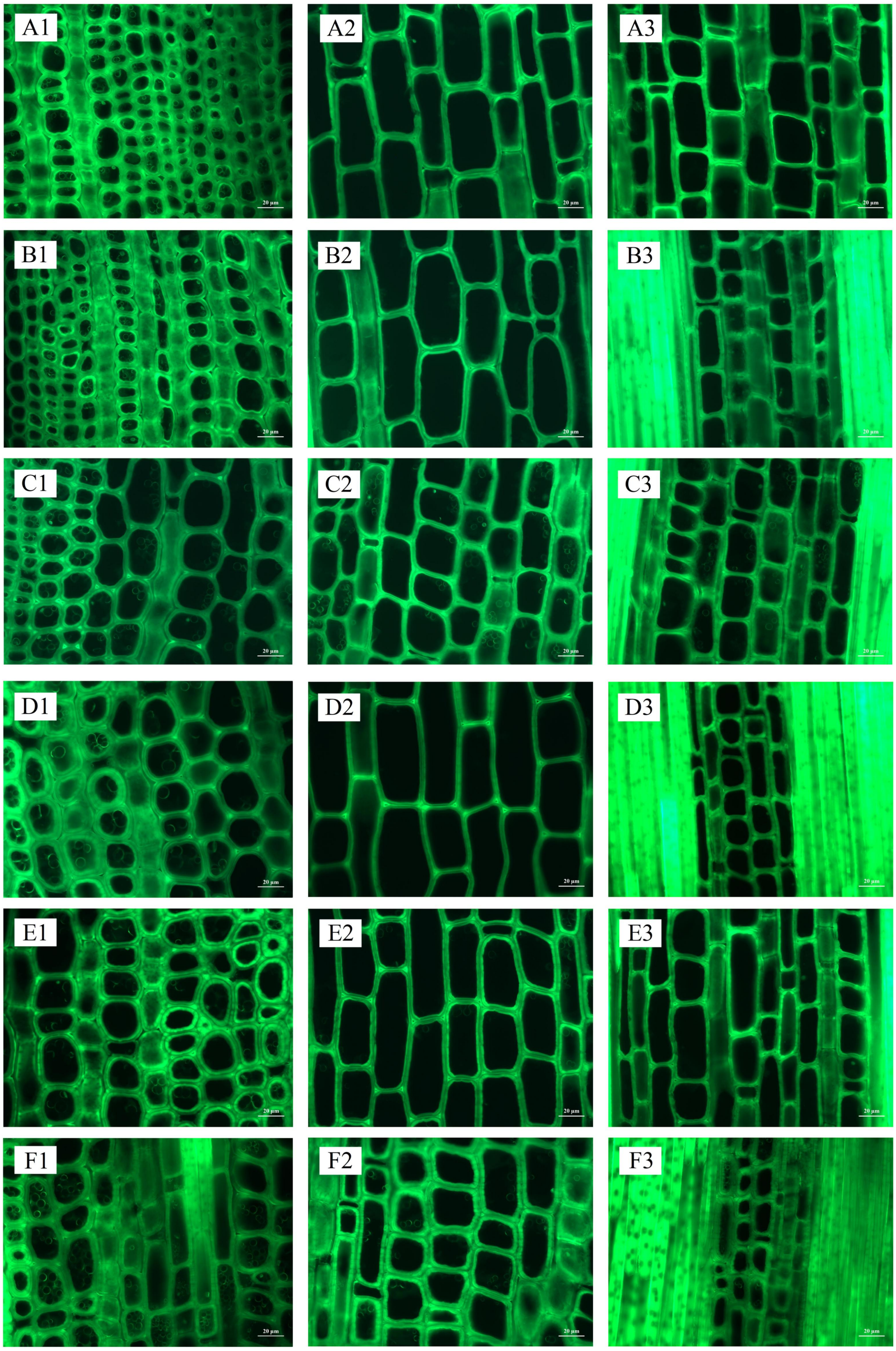

Figure S7. Distribution of starch granules in 5-year-old and 7-year-old bamboo culms in spring. (**A1**) The inner part of the base internode of 5-year-old culm; (**A2**) The middle part of the base internode of 5-year-old culm; (**A3**) The outer part of the base internode of 5-year-old culm; (**B1**) The inner part of the internode at breast height of 5-year-old culm; (**B2**) The middle part of the internode at breast height of 5-year-old culm; (**B3**) The outer part of the internode at breast height of 5-year-old culm; (**C1**) The inner part of the upper internode of 5-year-old culm; (**C2**) The middle part of the upper internode of 5-year-old culm; (**C3**) The outer part of the upper internode of 5-year-old culm; (**D1**) The inner part of the base internode of 7-year-old culm; (**D2**) The middle part of the base internode of 7-year-old culm; (**D3**) The outer part of the base internode of 7-year-old culm; (**E1**) The inner part of the internode at breast height of 7-year-old culm; (**E2**) The middle part of the internode at breast height of 7-year-old culm; (**E3**) The outer part of the internode at breast height of 7-year-old culm; (**F1**) The inner part of the upper internode of 7-year-old culm; (**F2**) The middle part of the upper internode of 7-year-old culm; (**F3**) The outer part of the upper internode of 7-year-old culm.

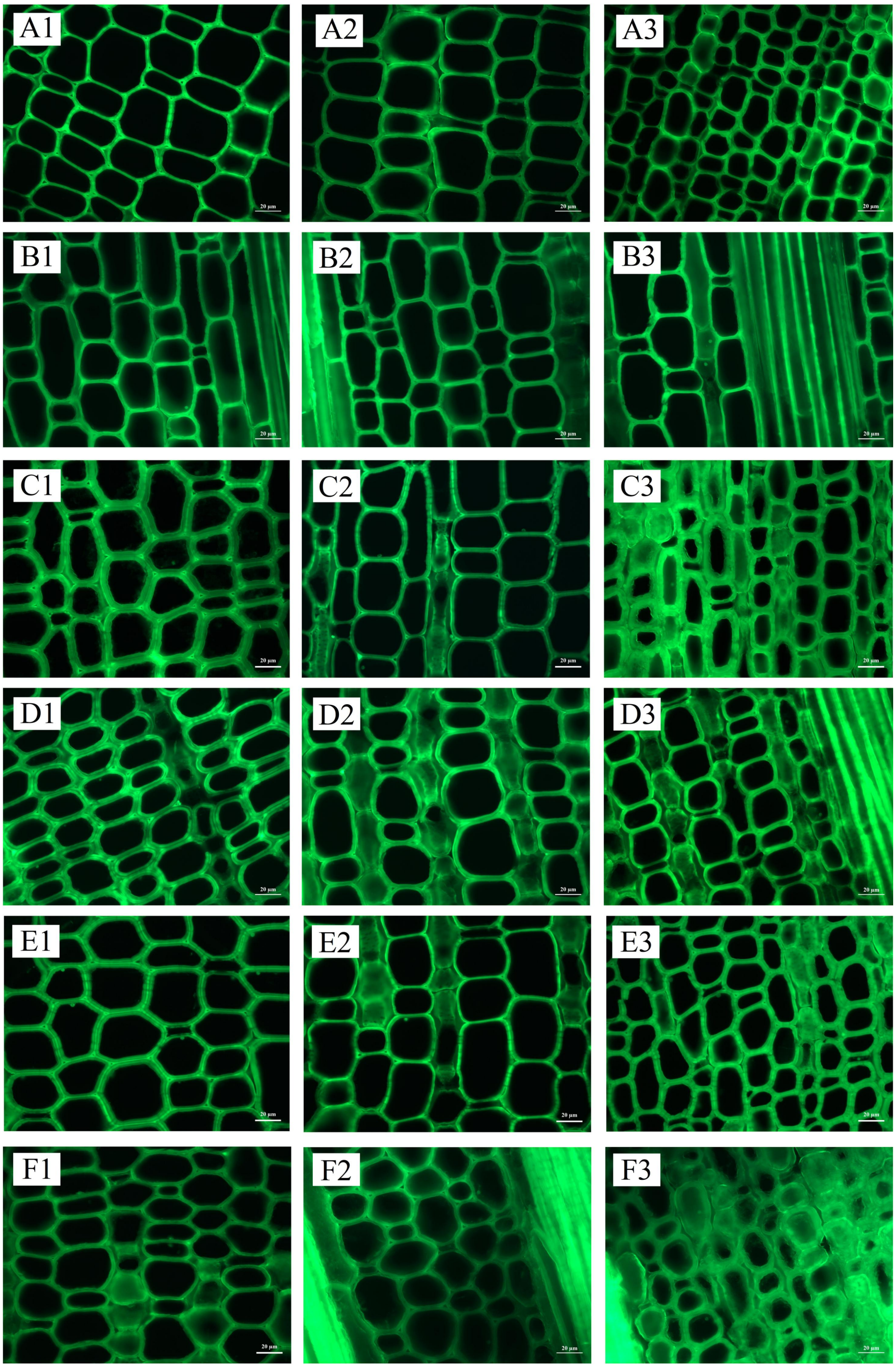

Figure S8. Distribution of starch granules of the rhizome connected with 1-3 years old bamboo culms in winter. (**A1**) The inner part of the coming rhizome connected with 1-year-old culm; (**A2**) The middle part of the coming rhizome connected with 1-year-old culm; (**A3**) The outer part of the coming rhizome connected with 1-year-old culm; (**B1**) The inner part of the going rhizome connected with 1-year-old culm; (**B2**) The middle part of the going rhizome connected with 1-year-old culm; (**B3**) The outer part of the going rhizome connected with 1-year-old culm; (**C1**) The inner part of the coming rhizome connected with 2-year-old culm; (**C2**) The middle part of the coming rhizome connected with 2-year-old culm; (**C3**) The outer part of the coming rhizome connected with 2-year-old culm; (**D1**) The inner part of the going rhizome connected with 2-year-old culm; (**D2**) The middle part of the going rhizome connected with 2-year-old culm; (**D3**) The outer part of the going rhizome connected with 2-year-old culm; (**E1**) The inner part of the coming rhizome connected with 3-year-old culm; (**E2**) The middle part of the coming rhizome connected with 3-year-old culm; (**E3**) The outer part of the coming rhizome connected with 3-year-old culm; (**F1**) The inner part of the going rhizome connected with 3-year-old culm; (**F2**) The middle part of the going rhizome connected with 3-year-old culm; (**F3**) The outer part of the going rhizome connected with 3-year-old culm.

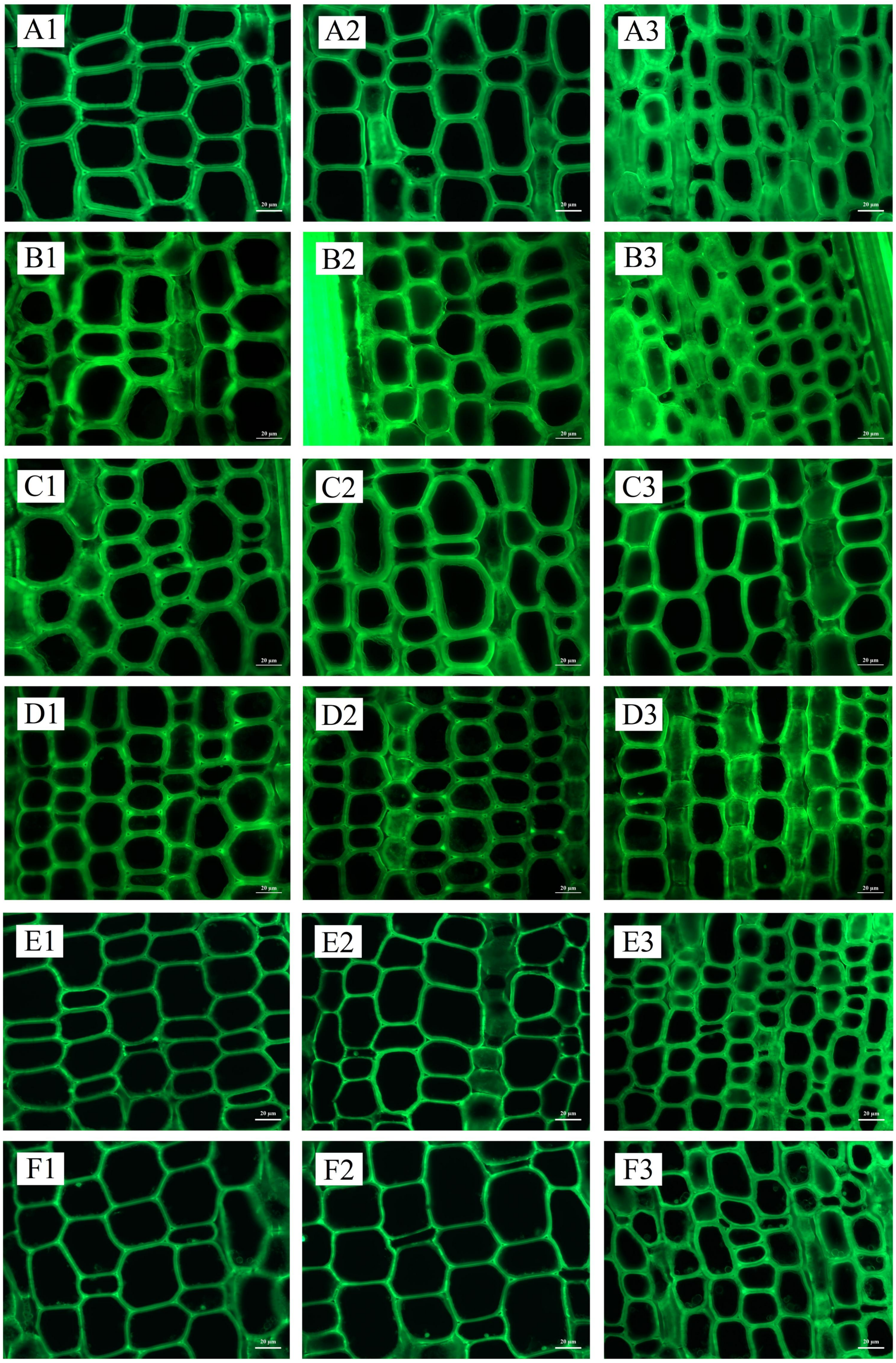

Figure S9. Distribution of starch granules of the rhizome connected with 4, 5, and 7-year-old bamboo culms in winter. (**A1**) The inner part of the coming rhizome connected with 4-year-old culm; (**A2**) The middle part of the coming rhizome connected with 4-year-old culm; (**A3**) The outer part of the coming rhizome connected with 4-year-old culm; (**B1**) The inner part of the going rhizome connected with 4-year-old culm; (**B2**) The middle part of the going rhizome connected with 4-year-old culm; (**B3**) The outer part of the going rhizome connected with 4-year-old culm; (**C1**) The inner part of the coming rhizome connected with 5-year-old culm; (**C2**) The middle part of the coming rhizome connected with 5-year-old culm; (**C3**) The outer part of the coming rhizome connected with 5-year-old culm; (**D1**) The inner part of the going rhizome connected with 5-year-old culm; (**D2**) The middle part of the going rhizome connected with 5-year-old culm; (**D3**) The outer part of the going rhizome connected with 5-year-old culm; (**E1**) The inner part of the coming rhizome connected with 7-year-old culm; (**E2**) The middle part of the coming rhizome connected with 7-year-old culm; (**E3**) The outer part of the coming rhizome connected with 7-year-old culm; (**F1**) The inner part of the going rhizome connected with 7-year-old culm; (**F2**) The middle part of the going rhizome connected with 7-year-old culm; (**F3**) The outer part of the going rhizome connected with 7-year-old culm.
